# The diagnostic and treatment values of double-balloon enteroscopy in children's Meckel's diverticular bleeding

**DOI:** 10.1097/MD.0000000000024823

**Published:** 2021-03-12

**Authors:** Zhongsheng Zhu, Shaoming Zhou, Huabo Cai, Hongyan Zhao, Zhaoxia Wang

**Affiliations:** Department of Gastroenterology, Shenzhen Children's Hospital, Shenzhen city, Guangdong Province, China.

**Keywords:** bleeding, children, double-balloon enteroscopy, Meckel's diverticulum, safety, surgical operation

## Abstract

The diagnostic and treatment values and safety of preoperative double-balloon enteroscopy (DBE) for Meckel's diverticula (MD) bleeding in children by retrospective review and analyses.

The clinical data were collected and analyzed from 10 cases of children with MD receiving preoperative DBE examination and postoperative pathological confirmation. The diagnostic and treatment values and safety were assessed through the comparison of the DBE results and intra-operative observations and subsequently postoperative pathological results.

Total cases are 10, 7 males and 3 females. The male to female ratio is 2.3 to 1. The youngest patient is 3.3 years old and oldest 12.1, the average age is 7.4 ± 3.0. The lowest body weight is 12.6 kg and the average is 32.5 ± 18.9 kg. The hematochezia was the main clinical manifestation in all patients with anemia and moderate to severe anemia were common (9/10, 90%). All patients had and tolerated the DBE procedures via anal route with 100% success rate. There were no observable complications during the examinations and post operations. All patients were diagnosed with MD by DBE. Exploratory laparoscopy and surgical operations were subsequently performed. All surgical samples were confirmed by pathology as bleeding MD. The postoperative follow-ups up to April 2019 (from 3 to 12 months) do not show any bleeding sign. Pathological examinations found ectopic gastric mucosa in 9 patients (90%) and one case had both ectopic gastric mucosa pancreatic tissue (10%). The distance of MD to ileocecal valve was from 60 to 100 cm (average 81.0 ± 16.0 cm) by DBE examinations. Surgery showed similar findings from 30 to 100 cm (average 71.0 ± 18.5) consistently to DBE. There is no statistical significance between 2 methods (Ζ = 1.715, *Ρ* = .086).

DBE examination proves to be a safe method for diagnosing children's MD disease and can reliably determine the bleeding lesions in children's MD, providing valuable guidance for surgical treatment of children's MD bleeding.

## 1 Introduction

Meckel's diverticulum, MD, also known as distal ileal diverticulum, is one of the most common congenital anomalies in the gastrointestinal tract. MD generally does not show clinical symptoms and only manifests when complications occur. The symptoms resulting from complications are often severe and require surgical treatment.^[1]^ Gastrointestinal bleeding is the most common complication.^[2]^

It is often challenging to diagnose children's MD before surgery with current available methods. Conventional non-invasive diagnostic methods such as radiography, angiography, ultrasound and CT prove to be of little value in diagnosis of MD.^[3]^ Tc^99m^-Pertechnetate scan is currently the standard and non-invasive method in diagnosing MD with 89.7% sensitivity and 92.9% specificity.^[4]^ However, it can sometimes show false negativities. If the scan does not show presence of MD and clinical symptoms highly suggest MD bleeding, it is necessary to perform Double-balloon enteroscopy (DBE) examination. In this study we retrospectively analyzed the clinical data from 10 children inpatients in the Gastroenterology Department of our hospital who received preoperative DBE examinations and were confirmed as MD bleeding by surgical operations and pathological examinations. Meckel scan was negative in all the 10 children included in this study. At the same time, all patients were examined with routine noninvasive methods, including electronic gastroscopy, radiology, ultrasound and CT scan. None of the methods pinpointed the bleeding location. Since they were highly suspected of MD bleeding, the DBEs were performed. The value and safety of preoperative laparoscopic DBE examination were assessed in diagnosing children's MD bleeding.

## 2 Data and methods

### 2.1 General data

All data were collected from 10 children inpatients in our Gastroenterology Department who suffered gastrointestinal bleeding and were suspected of MD bleeding by preoperative DBE and subsequently confirmed by intraoperative and postoperative pathology. Among them 7 are males and 3 females with average age of 7.4 ± 3.0 years. The duration of symptoms lasted from half a day to 6 months. Bloody stool was the common clinical symptom, dark or bright red in color. Four children had stomach discomfort or minor pain. All children showed anemia to various degree. The lowest hemoglobin was 52 g/L with average of 67.3 ± 14.1 g/L. All patients were examined with routine noninvasive methods, including Meckel scan, electronic gastroscopy, radiology, angiography, ultrasound and CT scan. None of the methods pinpointed the bleeding location. Since they were highly suspected of MD bleeding, the DBEs were performed with parents’ or guardian's permission. We refered to the operating procedures, indications and contraindications of China Guidelines of Clinical Applications for Small Intestinal Endoscopy in 2018, and combined with the characteristics of children, all children were given drug treatment to control bleeding before DBE examination, and DBE examination was performed after 2 to 3 days of stable condition and no active bleeding.

This study was approved by the Institutional Ethics Committee of the Shenzhen Children's Hospital. Written informed consent was given by the caregiver of children for their clinical records to be used in this study.

### 2.2 Operation methods

#### 2.2.1 Instrument

DBE system is from Fuji Corporation, Japan, model EN-530T. This system includes 4 parts: the main unit, endoscope, outer tubing and pump. The endoscope has 200 cm working length with 9.3 mm outer diameter.

#### 2.2.2 Preparations for DBE procedure

Comprehensive evaluations on patient's history and physical examination were performed and full preoperative laboratory work was done including blood routine examination, liver and kidney functions, myocardial zymogram, blood coagulation function, hepatitis B and C, HIV, syphilis antibody, cardiography, chest x-ray etc. All patients did not have absolute clinical contradictions. Written permissions were signed by patient's guardians. Preoperative preparation procedures refer to 2013 China Guideline on Bowel Preparation for Digestive Endoscopy (Draft).^[5]^ Specifically 1 day before the procedure either low-fiber or liquid food is given or no food is allowed after dinner. Four to Six hours before the examination Polyethylene Glycol Electrolytes Powder II (Wanhe, Shenzhen, SFDA Approval No. H20030827) is administrated via oral route completing within 2 hours. No water is allowed 2 hours before the examination. To the patients who could not take in sufficient amount of Polyethylene Glycol Electrolytes Powder II, Nasogastric tube dripping was used.^[6]^ For a few uncooperative patients, intestinal lavage with saline solution was administrated until there were no solids in the feces. All children patients were deeply sedated and generally anesthetized by an anesthetist. They were monitored through the whole procedure with their heart rate, oxygen saturation and blood pressure, etc.

#### 2.2.3 Operating procedures

After taking into account of patient's history, bleeding characteristics and other diagnostic examinations (suggesting MD bleeding), the anal route was decided as first choice. According to the operating procedures from China Guidelines of Clinical Applications for Small Intestinal Endoscopy, the patients were laid down on their left side and the operator held the endoscope with the left hand and advanced with the right hand. When the endoscope approached the border between sigmoid and descending colons, the air sac in front of the endoscope was inflated first to secure the endoscope from moving around. Subsequently, the outer tube was slid in around the endoscope body, followed by inflation of the air sac in front of the outer tube. At this stage, both air sacs were inflated and the position was fixed among the endoscope, outer tube and the intestinal wall, the endoscope and outer tube were pulled to make sigmoid colon straight. Next the air sac in front of the endoscopy was deflated and the endoscopy was advanced to splenic flexure of colon. By repeating these steps, the endoscope reached hepatic flexure of colon and ileocecal valve thereafter. While at the ileocecal valve, the endoscope was pushed into ileal terminus first, followed by air sac inflation, fixation of the endoscopy, and advancement of outer tube and its air sac inflation. By repeating above mentioned inflation, deflation and pull-push steps, the endoscope was pushed as deep as possible into the small intestine. The procedure stopped once the lesion was found.^[7]^

#### 2.2.4 Endoscopic depth and distance of lesion location by using distance accumulation^[8]^

These were calculated by adding up all valid advancement of endoscope, which was through recording the advancement distance of the endoscope body in every round (A) and subtracting the distance from slip off or invalid advancement (B). So the depth=(A1-B1) + (A2-B2) + … +(An-Bn).

#### 2.2.5 MD diagnosis

Two tubular structures were observed by DBE and 1 was normal intestine and the other was a blind end structure in which mucosa and blood vessels were often seen. The final diagnosis was confirmed by surgery and postoperative pathology.

### 2.3 Statistics

Data were analyzed using SPSS version 19.0. Continuous variables were represented with mean±SD unless otherwise indicated. Categorical variables ere represented with frequency analysis n (%). The paired comparison was performed using Wilcoxon test and significant if *P* < .05.

## 3 Results

### 3.1 Completion of DBE examination

All 10 children patients completed their DBE procedures with 100% success (10/10). All patients received their examination via anal routes (*tans* method). The measurements from MD to ileocecal valve were 60 to 100 cm with average of 81.0 ± 16.0 cm.

### 3.2 Clinical manifestations of the patients

In the 10 cases, there were 7 males and 3 females (2.3:1). The ages showing clinical symptoms were from 3.3 to 12.1 years old, averaging 7.4 ± 3.0. The lowest body weight of the patients is 12.6 kg with average of 32.5 ± 18.9 kg. They showed differences in their initial symptoms: all 10 presented hematochezia, and among them 6 discharged dark-red stool, 2 scarlet-red and 2 tarry-red. Five of them showed accompanying symptoms in which 2 experienced abdominal discomfort and pain, 1 had low fever and 2 vomited. 9 patients bled for the first time and 1 repeatedly 4 times. All patients manifested anemia to various degree with hemoglobin of 52 to 101 g/L, averaging 67.3 ± 14.1 g/L and majority showed moderate to severe anemia. Nine were given blood transfusion and 1 of them was given twice. See Table 1 for details.

**Table 1:** Clinical features of Patients with MD observed by DBE.

Features	Cases(n)	Percentage (%)
Gender		
Male	7	70
Female	3	30
Age		
3–6	4	40
6–12	5	50
12–	1	10
Symptoms		
hematochezia	10	100
Abdominal discomfort or pain	2	20
Vomiting	2	20
Low fever	1	10
Anemia		
Mild	1	10
Moderate	5	50
Severe	4	40

### 3.3 The characteristics of lesions under endoscope and surgery

**Figure 1:**
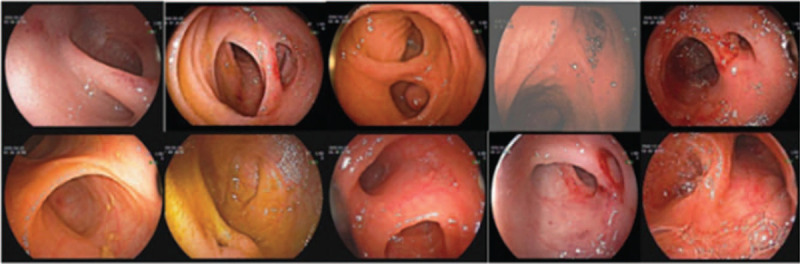
Endoscopic features of MD.

All patients were found the presence of MD by DBE. The typical representation was that there were 2 tubular structures, one of them was the small intestine and the other was a blind end structure. The abnormal structure showed mucosal congestion and edema and some also showed mucosal erosion and bleeding (Fig. 1). After DBE examination all patients underwent laparoscopic operation. Based on the base widths and the extent of intestinal stenosis, either the wedge diverticulum resection (5) or partial ileal resection and end-to-end anastomosis (5) were performed. It was found during the surgery that the lengths of MD were between 1.5 and 6.0 cm with average of 3.5 ± 1.3 cm; the widths between 1.0 and 3.0 cm with average of 2.1 ± 0.9 cm (Table 2). It was also found in surgery that the distances of MD from the ileocecal valve were between 30 and 100 cm with average of 71.0 ± 18.5 cm. The difference between the measurements from DBE and surgery were statistically insignificant (*P* = .086) (Table 3).

**Table 2:** Clinical features of patients with MD in surgery.

Items	Cases(n)	Percentage (%)
Operation methods		
wedge diverticulum resection	5	50
partial ileal resection and end-to-end anastomosis	5	50
MD Features		
Length (cm)		
1∼3	5	50
3∼6	5	50
Diameter (cm)		
∼1	3	30
1∼3	7	70
Atypical tissues		
Gastric mucosa	9	90
Pancreatic tissues	0	0
Both	1	10

**Table 3:** Comparison of distances (cm) of MD from ileocecal valve between DBE and surgery.

Case#	DBE (cm)	Surgery (cm)
1	70	80
2	80	80
3	60	30
4	100	70
5	60	60
6	100	100
7	70	80
8	80	60
9	90	80
10	100	70

Z = 1.715, *P* = .086

### 3.4 The safety of DBE examination

All 10 children patients tolerated the DBE examination to completion. There were no severe complications such as intestinal bleeding, perforation, tearing of the intestinal mesenteric root tissue and secondary infection or anesthetic complications during the DBE examination and after surgery. One patient experienced mild postsurgery abdominal discomfort due to the inflation of the examination. One had anal discomfort after surgery. Their symptoms receded naturally after rest.

### 3.5 Pathological results

**Figure 2:**
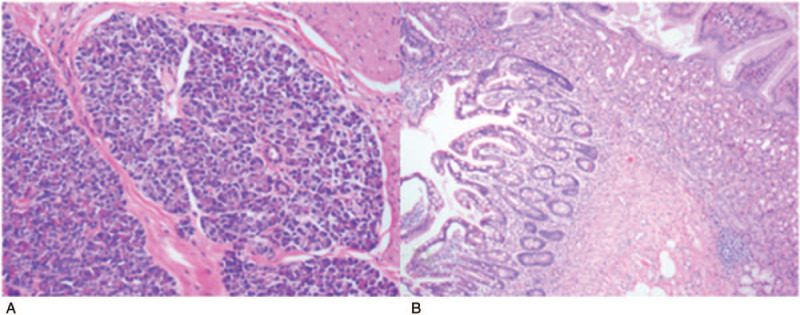
Pathological features of MD: A. Atypical Pancreatic tissue B. Atypical gastric tissue.

All surgical samples were pathologically examined and confirmed to be MD. All 10 cases were found to have atypical mucosal tissues inside the MD, 9 (90%) were gastric mucosa and 1 (10%) additionally had pancreatic tissue (Fig. 2). Because bowel preparation must be carried out before DBE examination, combined with the characteristics of children and the safety of DBE examination, all children were given drug treatment before DBE examination, and the bleeding was completely controlled before DBE examination. Therefore, no obvious active bleeding was found in the diverticulum after DBE examination. Only congestion and edema of intestinal mucosa were found in the diverticulum, and erosion or a small amount of bleeding were found in the diverticulum, 5 of them were located at the bottom of the diverticulum and 3 cases were located around the outer mouth of the diverticulum.

### 3.6 Follow-up

All patients were followed up after discharged by phone or outpatient visits for 3 to 12 months (until April 2019) and none of them were found to be bleeding.

## 4 Discussion

Meckel Diverticulum (MD) results from abnormal development of the vitelline duct during embryogenesis. Approximately at the end of 5th week of embryogenesis when the placental circulation is formed, the vitelline duct starts to shrink into a fibrous cord subsequently is absorbed from the umbilical end to the intestinal end. However, if the umbilical end is absorbed and the intestinal end is not, the latter will form a blind end sac, namely, ileal distal diverticulum.^[1]^ Meckel first in 1809 reported this congenital deformation and provided a detailed description of its embryology and clinical characteristics, hence it was named as Meckel diverticulum (MD).^[9]^ The morbidity of MD generally is around 2% with more males than females.^[10]^ It was reported that the ratio of males to females is between 2 to 1 and 4 to 1.^[11]^ In our current study the average age of children patients is 7.4 ± 3.0 years old and the ratio is 2.3 to 1, consistent with the literature.

MD clinically manifests in a great diversity and lacks specific symptoms and signs. The majority of the patients are asymptomatic and only show clinical symptoms when complications occur such as bleeding, perforation, stenosis and diverticulitis. MD is the remnant of the vitelline duct in which tissue cells possess the ability to differentiate in various mucosae. Therefore it is common to find atypical mucosal tissues inside the diverticulum, mostly gastric mucosa, pancreatic tissue and intestinal mucosa.^[12]^ They can secrete chloric acid and digestive enzymes causing ulceration, bleeding and even perforation inside the diverticulum and adjacent tissues.^[13]^ In our study, the pathological examination found that all the patients had atypical gastric mucosa with 1 accompanying pancreatic tissue. Lin et al also reported that the lower gastrointestinal bleeding is the most common clinical manifestations in children's MD.^[14]^ The children patients in this study showed hematochezia as their main complaint without other evident prodromal symptoms. There was 1 case accompanying with low fever, 2 with vomiting, and other 2 abdominal discomfort or pain but not severe. Most patients (9/10, 90%) showed severe lower gastrointestinal bleeding and required blood transfusion. Hence the early diagnosis is vital to the patients.

MD bleeding is the most common cause in children with low gastrointestinal bleeding. Once diagnosed surgical operation is required. Since the lesions locate in ileum, the preoperative diagnosis is challenging. Routine noninvasive diagnostic methods such as radiography, angiography, ultrasound and CT have little value in diagnosis.^[3]^ It is reported that most MD is often situated over 60 cm above the ileocecal valve,^[15]^ which makes it difficult for traditional gastroscope or colonoscope to reach the site. Tc^99m^-Pertechnetate scan proves to be specific to MD with atypical gastric mucosa but often shows false positivity for those with small diverticulum, without or with a little atypical gastric mucosa or with severe inflammation affecting the isotope absorbance.^[16–17]^ DBE, aided by air sacs, can lead the endoscope deep into the small intestine thus fulfilling the diagnosis and treatment of MD.^[18]^ Leung et al in 2007 first reported the application of DBE in children patients.^[19]^ Since then DBE application has gradually increased in diagnosing children's small intestinal diseases.

In this study all patients were found absent of MD by noninvasive methods but highly suspected of MD clinically. By DBE examination they were found suffering from MD, which were consequently confirmed to be MD bleeding by surgery and postoperative pathological examination. All children received DBE via anal route and went through the whole procedure until the lesions were found. There were no serious complications during DBE examination or after surgery. The distances between MD site to ileocecal valve measured by DBE was 60 to 100 cm (mean ± SD=81.0 ± 16.0 cm), while surgery found that the distances were 30 to 100 cm (mean ± SD =71.0 ± 18.5). These findings are consistent to that reported by Geng et al.^[20]^ There is no statistical difference between DBE and surgical measurements. Therefore DBE can accurately locate the MD bleeding sites and reliably provide guidance for surgical treatment of MD bleeding. Although Leung et al^[19]^ suggests that DBE examination is suitable for children over 30lb (14 kg), we successfully performed the procedure in children with body weight of 12.6 kg.

Laparoscopically surgical removal of MD is proven to be safe and effective in symptomatic patients and causing less traumatic, rarely severe complications and leading to rapid postoperative recovery.^[14]^ DBE confirmation in both diagnosis and location of MD greatly improves the safety and effectiveness of surgical operations. Due to the limitation of equipment and conditions, Meckel's diverticulum was not resected under endoscopy in our hospital. All patients were resected by laparoscopy after DBE examination. All 10 cases of children patients in this study underwent either wedge diverticulum resection or partial ileal resection and end-to-end anastomosis and fully recovered. They had no any complications and recurrent bleeding in long term follow-ups.

## 5 Conclusions

In summary, DBE examination can be safely and effectively applied to children patients. It is a reliable diagnostic method to children patients with MD bleeding in complement to other diagnostic techniques. It also plays an important guiding role in MD bleeding surgery.

## Acknowledgment

Special thanks to Dr Wenming Duan for her involvement in statistical analysis and review.

## Author contributions

**Conceptualization:** Zhongsheng Zhu, Zhaoxia Wang.

**Data curation:** Zhongsheng Zhu, Huabo Cai, Hongyan Zhao.

**Formal analysis:** Zhaoxia Wang.

**Funding acquisition:** Zhongsheng Zhu.

**Investigation:** Zhongsheng Zhu.

**Methodology:** Zhongsheng Zhu.

**Project administration:** Zhongsheng Zhu.

**Resources:** Zhongsheng Zhu.

**Software:** Zhongsheng Zhu.

**Supervision:** Zhongsheng Zhu, Shaoming Zhou.

**Validation:** Huabo Cai.

**Visualization:** Zhongsheng Zhu.

**Writing – original draft:** Zhongsheng Zhu.

**Writing – review & editing:** Zhaoxia Wang.
